# Steroid binding to Autotaxin links bile salts and lysophosphatidic acid signalling

**DOI:** 10.1038/ncomms11248

**Published:** 2016-04-14

**Authors:** Willem-Jan Keune, Jens Hausmann, Ruth Bolier, Dagmar Tolenaars, Andreas Kremer, Tatjana Heidebrecht, Robbie P. Joosten, Manjula Sunkara, Andrew J. Morris, Elisa Matas-Rico, Wouter H. Moolenaar, Ronald P. Oude Elferink, Anastassis Perrakis

**Affiliations:** 1 Division of Biochemistry, Netherlands Cancer Institute, 1066 CX Amsterdam, The Netherlands; 2 Department of Hepatology and Gastroenterology, Tytgat Institute for Liver and Intestinal Research, Academic Medical Center, University of Amsterdam, Amsterdam, The Netherlands; 3 Division of Cardiovascular Medicine, Gill Heart Institute, University of Kentucky, Lexington, Kentucky 40511, USA; 4 Department of Veterans Affairs, Medical Center Lexington, Kentucky 40511, USA; 5 Division of Cell Biology, Netherlands Cancer Institute, 1066 CX Amsterdam, The Netherlands

## Abstract

Autotaxin (ATX) generates the lipid mediator lysophosphatidic acid (LPA). ATX-LPA signalling is involved in multiple biological and pathophysiological processes, including vasculogenesis, fibrosis, cholestatic pruritus and tumour progression. ATX has a tripartite active site, combining a hydrophilic groove, a hydrophobic lipid-binding pocket and a tunnel of unclear function. We present crystal structures of rat ATX bound to 7α-hydroxycholesterol and the bile salt tauroursodeoxycholate (TUDCA), showing how the tunnel selectively binds steroids. A structure of ATX simultaneously harbouring TUDCA in the tunnel and LPA in the pocket, together with kinetic analysis, reveals that bile salts act as partial non-competitive inhibitors of ATX, thereby attenuating LPA receptor activation. This unexpected interplay between ATX-LPA signalling and select steroids, notably natural bile salts, provides a molecular basis for the emerging association of ATX with disorders associated with increased circulating levels of bile salts. Furthermore, our findings suggest potential clinical implications in the use of steroid drugs.

## Abstract (web summary)

Autotaxin generates the bioactive lipid lysophosphatidic acid to regulate diverse biological processes. Here, the authors identify a role for bile salts as direct allosteric inhibitors of autotaxin activity, suggesting that steroids may function as regulators of lysophosphatidic acid signalling.

## Graphical abstract

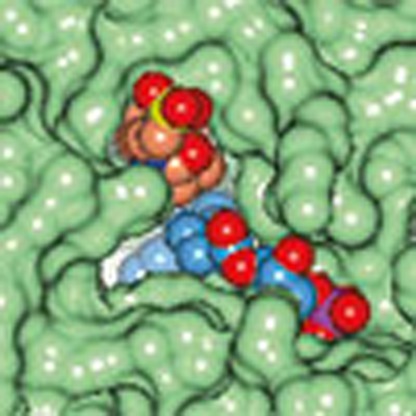

Autotaxin (ATX) is a secreted lysophospholipase D (lysoPLD) that produces the bioactive lipid lysophosphatidic acid (LPA) from extracellular lysophospholipids, predominantly lysophosphatidylcholine (LPC)^1^,^2^. ATX is a unique member of the ectonucleotide pyrophosphatase/phosphodiesterase (ENPP) family of enzymes that hydrolyse phosphodiester bonds in various substrates, including nucleoside triphosphates, lysophospholipids and choline phosphate esters^3^.

The bioactive product of ATX, LPA, acts on six distinct G protein-coupled receptors (LPA_1–6_) that activate multiple signalling pathways^4^. The biological outcome of ATX-LPA signalling is remarkably diverse, depending on LPA receptor expression patterns and tissue context, and includes the stimulation of cell migration, proliferation and survival^4^,^5^,^6^. In pathophysiology, ATX-LPA signalling has been implicated in tumour progression, pulmonary fibrosis, neuropathic pain, cardiovascular disease and cholestatic pruritus, making the ATX-LPA signalling axis an attractive therapeutic target^7^.

Crystal structures of ATX^8^,^9^ (ENPP2) revealed a catalytic domain with a bimetallic active site adjacent to a catalytic threonine. Substrate binding takes place in a shallow hydrophilic groove that accommodates nucleotides as well as the glycerol moiety of lysophospholipids, and in a deep hydrophobic pocket binding the lysophospholipid acyl chain. In addition, the catalytic domain together with the first of two somatomedin-β (SMB) domains, form a tunnel adjacent to the active site (Fig. 1a,b).

The function of this unique tunnel, which is absent in other ENPP family members like ENPP1 (refs 10, 11), remains enigmatic. One hypothesis based on structural and mutagenesis data suggests that the tunnel serves to deliver LPA to its cognate G protein-coupled receptors (GPCRs)^9^. Recent structural analysis shows that access to the LPA_1_-binding pocket occurs from the extracellular space^12^, in contrast to the proposed access route of the related lipid mediator sphingosine 1-phosphate (S1P) to its receptor S1P_1_ (ref. 13). ATX isoforms have affinity for cell-surface integrins^8^ and heparan sulphate proteoglycans^14^. In addition, kinetic analysis revealed that the half-time of the ATX:LPA complex is several minutes^15^, allowing ATX:LPA to diffuse and possibly recognize cell surface receptors, bringing the LPA product closer to the cell membrane. Collectively, these observations place ATX at the centre of an intrinsic network of interactions that could explain its extensive roles in diverse physiological and disease processes. Until now, no physiological regulators of ATX activity have been identified.

Our on-going studies of ATX structure and catalytic mechanisms reveals that recombinant ATX contained steroids bind to the tunnel. We discover that binding of at least one class of steroids, bile salts, modulates ATX activity. Our findings reveal an unexpected novel interplay between LPA and bile salts, and possibly other steroids, and offer testable hypotheses on the function of the ATX-LPA signalling axis in various physiological contexts.

## Results

### ATX binds steroids in its tunnel

**Figure 2:**
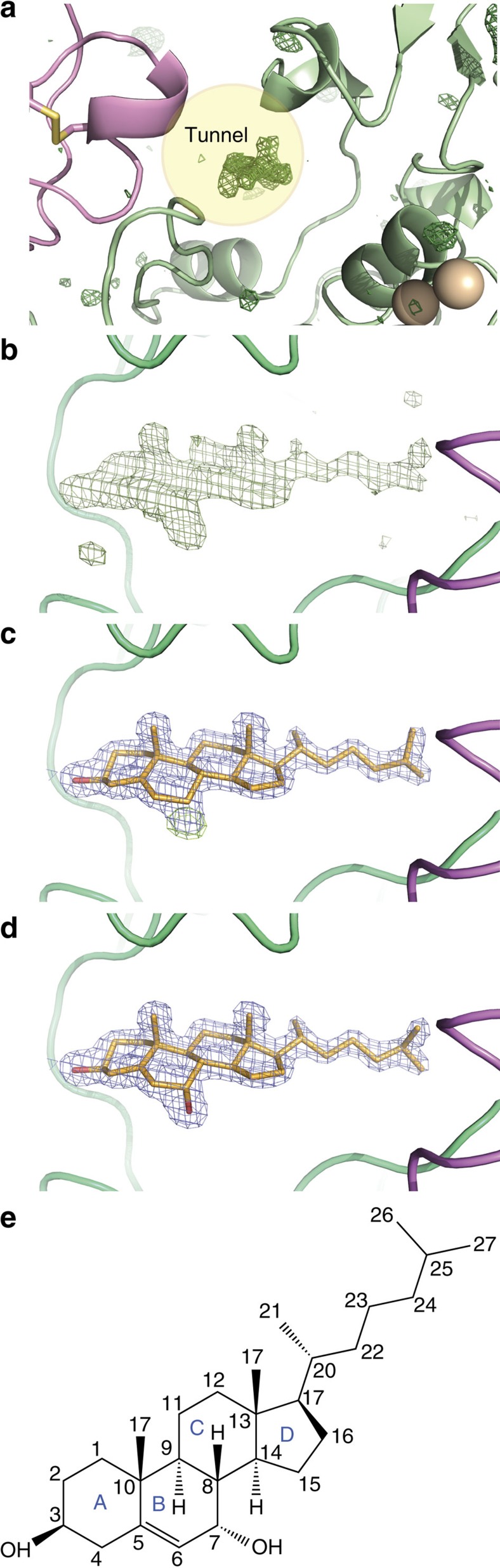
7α-OH steroids are bound in the tunnel of ATX. (**a**) A zoom-in to the model with difference density (*mF*_*o*_*−DF*_*c*_) shown as a green wireframe model before any ligand or water placement contoured at 3.5 RMS highlighting the unexplained density in the tunnel. (**b**) The model is rotated about 90° along the vertical display axis to show the same difference density map. (**c**) The model is shown in the same orientation as before after including a sterol molecule in the refinement; the resulting *2mF*_*o*_*−DF*_*c*_ map is shown in blue at 1.2 RMS and the difference map in green still indicates a missing atom. (**d**) The electron density maps after full refinement including the 7α-hydroxycholesterol molecule; all difference density peaks have disappeared. (**e**) The molecular structure of 7α-hydroxycholesterol showing all atom and ring names.

During the course of our structural investigation of the ATX catalytic mechanism, we obtained a 1.6-Å resolution structure of ATX (Table 1). Following structure refinement, some residual density in the tunnel became apparent (Fig. 2a,b). The density was compatible with the four-ring system bearing two axially oriented methyl groups, characteristic of steroid moieties. Indeed, modelling of cholesterol in the density yielded a nearly perfect fit (Fig. 2c). To explain remaining difference density, adjacent to the C_7_ position of the B ring, we modelled 7α-hydroxycholesterol (7HCS, Fig. 2d,e).

Notably, when we determined the first ATX structure^8^ (2XR9) at 2.05 Å resolution, we have noted that ‘residual electron density occupies this tunnel, probably indicating the presence of a molecule that we were unable to model (possibly a lipid acquired from the growth medium)'. When we re-examined the maps corresponding to that structure, with the hindsight that the residual electron density could be 7HCS, it was indeed trivial to fit a 7HCS moiety to that residual density in the tunnel (Supplementary Fig. 1). This indicated that 7HCS has always been present in our protein preparations, and it is certainly a bound ligand in the 2XR9 ATX structure, which we were unable to identify at the time. Indeed, the presence of cholesterol (∼15%), hydroxysterols (∼80%) and 7HCS specifically (∼75% of total hydroxysterols and ∼60% of the total) at near stoichiometric levels to ATX, in several preparations, was confirmed by mass spectrometry (Table 2 and Supplementary Fig. 2). The mass spectrometry data also suggest that the predominant 7HCS species is the α epimer. The 7HCS structure and the revised 2XR9 structure strongly suggest that 7HCS is most likely the only bound molecule, albeit the exact nature of the aliphatic chain could not be uniquely identified.

The steroid moiety is oriented away from the active site with the 3-OH group facing the solvent on the opposing surface of the molecule. The steroid rings are well packed, with the conserved Trp254, Trp260 and Phe274 likely participating in π–π interactions. The 7-OH group is clearly only in a single anomeric configuration (*α*) as suggested by the mass spectrometry data; the preference is explained by a hydrogen bond to the Ν_ζ_ atom of Trp260. The selectivity towards the 7-OH substituted steroid is notable, as a Protein Data Bank (PDB) search suggests this is the only example of specific hydroxysterol recognition by a carrier protein.

### Bile salts bind ATX and inhibit LPA production

**Figure 3:**
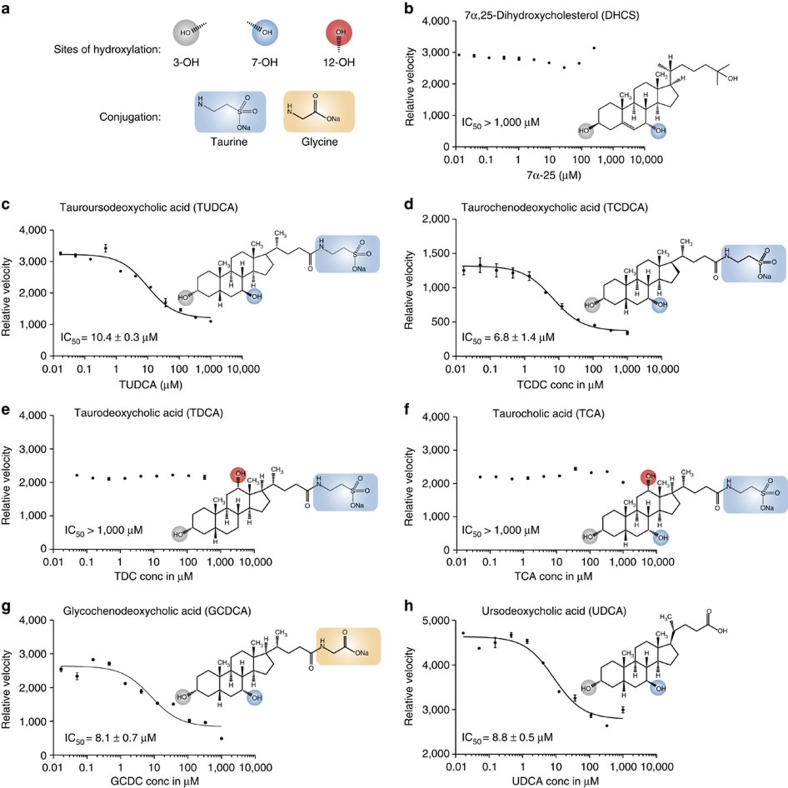
Activity of ATX in the presence of selected steroids. (**a**) A visual guide to the annotations used for indicating specific functional groups in the bile acids chemical formulas (**b**–**h**) Inhibition of lysoPLD ATX activity measured as released choline by LPC(18:1) hydrolysis in the presence of specific bile salts with different steroid moiety hydroxylation patterns and acidic tail conjugations. The 12-OH substitution is not tolerated while the conjugation does not affect activity. The grey, blue and red circles highlight the hydroxylation of the steroid backbone; the orange and blue boxes highlight the conjugation partner. The error bars represent s.e.m. from triplicate experiments. Conc, concentration.

As sterols were not present during ATX purification, while ATX was selective for a 7α-substituted steroid presumably acquired from the cells and/or cell culture medium, we went on to investigate how distinct steroids may modulate ATX activity (Fig. 3). Hydroxysterols like 7HCS or 7α,25-dihydroxycholesterol, as well as various other steroids (testosterone, dexamethasone, prednisolone), had no effect on ATX-mediated LPC hydrolysis (Fig. 3b and Supplementary Fig. 3). Strikingly, however, the conjugated bile salt tauroursodeoxycholate (TUDCA) inhibited ATX activity with an apparent IC_50_ of 10 μM (Fig. 3c). Structure–activity relationship studies using bile salts with different substitutions of the steroid rings and different conjugates of the acidic tail, revealed that: (i) bile salts with the C_7_ hydroxyl in the α-configuration (taurochenodeoxycholate) or in the β-configuration (TUDCA) inhibit ATX activity with similar potency (Fig. 3 c,d); (ii) the presence of a hydroxyl in C_12_ of the C ring, either in the presence of the C_7_ hydroxyl (that is, taurocholate) or in the absence of the C_7_ hydroxyl (that is, taurodeoxycholate) abrogate the inhibitory activity of TUDCA (Fig. 3e,f); and (iii) the conjugation of the acidic tail did not significantly change potency, as shown by using glycochenodeoxycholate (which is conjugated to glycine instead of taurine) or ursodeoxycholate (lacking conjugation; Fig. 3g,h).

**Figure 4:**
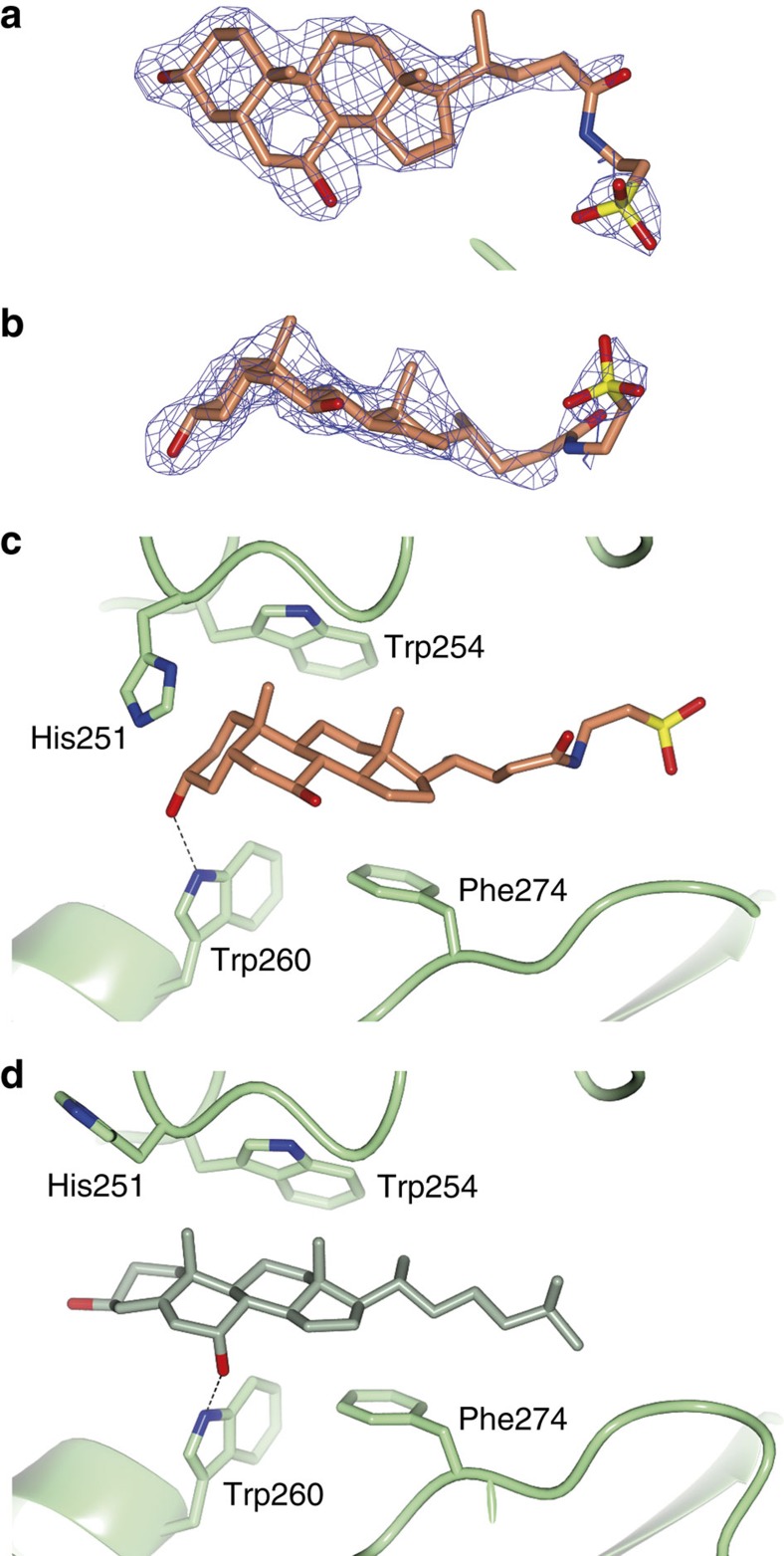
The bile salt TUDCA binds the tunnel of ATX. (**a**,**b**) The *2mF*_*o*_*-DF*_*c*_ electron density map before ligand placement is contoured at 1.2 RMS and shown as a blue wireframe model in two views; TUDCA (orange carbons; oxygens in red, sulfur in yellow and nitrogen in blue) is shown as a stick model. (**c**,**d**) Comparison of the binding of TUDCA and 7α-hydroxycholesterol; Trp260 forms a hydrogen bond (dotted line) with the 3α-OH of TUDCA (in orange), whereas it forms a hydrogen bond with 7α-OH of the hydroxycholesterol (in green). In the TUDCA-bound structure, His251 flips towards the A-ring, whereas in both structures Trp260 and Phe274 pack against the steroid ring system.

To understand the selectivity of bile salts on ATX activity, we co-crystallized ATX with TUDCA and determined the structure to 2.0 Å resolution (Table 1). Following molecular replacement and model adjustment, there was a clear difference electron density in the tunnel, markedly different from the density for 7HCS (Fig. 4a,b). Owing to the reduction of the C_5_–C_6_ double bond of the steroid B-ring, the four-ring system in bile salts is no longer planar, but adopts an L-shaped conformation (Fig. 4b). In comparison with the 7HCS placement, the steroid moiety of TUDCA has ‘slid' inside the tunnel by about 2.5 Å towards the active site (Fig. 4c,d). This movement ‘aligns' the D-ring better with Phe274, likely resulting to more favourable π–π interactions. The Ν_ζ_ of Trp260 now forms a hydrogen bond with the 3-OH group of the A-ring, instead of the 7-OH that has moved away. In addition, the conserved His251 flips towards the tunnel exit, acting as a ‘lid'. Overall, the ring placement indicates a stronger interaction of TUDCA compared with 7HCS in the tunnel. Given that the tunnel may serve as an LPA exit route^9^, this observation might provide a mechanism for the inhibitory activity of TUDCA. Unlike the steroid moiety, the acidic tail is not very well resolved in the electron density. Further refinement allowed modelling of three district conformations of the TUDCA acidic tail (Supplementary Fig. 4a,b).

Strikingly, structural alignment of the TUDCA structure with the 7HCS structure or the native structure (2XR9), showed that the SMB domains move ‘outwards' to accommodate TUDCA in the tunnel (Supplementary Fig. 4c); a movement of the ATX SMB domains relative to the catalytic core has not been observed before. Thus, TUDCA binding induces a conformation change, which may have implications for SMB domain interactions and binding site dynamics.

The observed multiple conformations of the acidic tail, contrasting with the well-defined binding of the bile salt steroid moiety, agrees well with the structure–activity data, and prompted us to examine further the inhibition mechanism of bile salts towards ATX.

### Bile salts are non-competitive inhibitors of LPC hydrolysis

**Figure 5:**
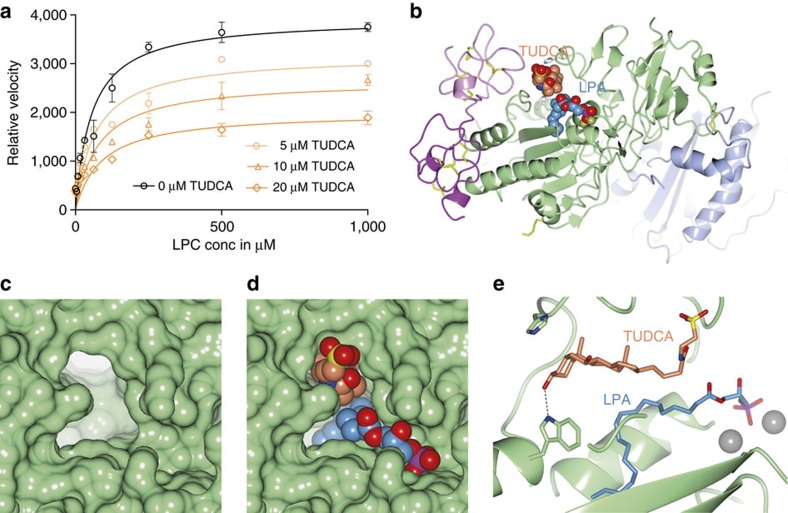
TUDCA acts as a non-competitive inhibitor of LPC hydrolysis. (**a**) ATX lysoPLD activity with no inhibitor (black line and symbols) and with three concentrations (conc) of TUDCA (orange lines and symbols) as function of LPC(18:1) substrate conc. Modelling of all data (see the Methods for details) indicate that TUDCA acts as a partial noncompetitive inhibitor, with a *K*_i_ of 9±3 μM and residual activity of ∼40% towards LPC. (**b**) A cartoon of the ATX structure with bound TUDCA (orange carbons) in the tunnel and LPA (blue carbons) in the pocket, both shown as space filling models. (**c**,**d**) A zoom-in view showing the molecular surface of ATX at the TUDCA- and LPA-binding sites empty (**c**) and with bound TUDCA and LPA (**d**) as space filling models. (**e**) A zoom-in to a view along the tunnel axis, showing the characteristic L-shaped bile acid ring system and the bound LPA(18:1); the taurine tail has moved away from the active site to make space for the LPA; the active site zincs are visible to the right as grey spheres.

Testing the inhibitory effect of different TUDCA concentrations against increasing concentrations of LPC, showed a reduction in *V*_max_ with no significant change in the *k*_M_ of the reaction (Fig. 5a); consistently, kinetic modelling confirmed that a competitive model has a probability of <0.01% to be correct (see the Methods for details and equations). As TUDCA is a partial inhibitor of ATX (Fig. 3b), we used a partial mixed inhibition model to explain the kinetic data (see the Methods for details). Kinetic modelling suggested that while TUDCA has an appreciable *ϰ*_I_ of 9±3 μM, even at saturating concentrations ∼40% of ATX activity towards LPC is retained. Crucially, mathematical modelling indicated that TUDCA is a noncompetitive inhibitor, implying that LPC or LPA can co-exist in a ternary complex with ATX.

To test this hypothesis, we crystallized a complex of ATX, TUDCA and LPA and we determined a structure of this ternary complex to 1.8 Å resolution (Table 1). In this complex, TUDCA is well defined in density (Supplementary Fig. 5) and the steroid moiety lies in the tunnel (Fig. 5b–d), but the conjugated tail is in a single well-defined conformation pointing away from the active site (Fig. 5e). LPA(18:1) binds in a similar pose as shown earlier for mouse ATX^9^ (3NKP); whereas the glycerol moiety is well defined in density, the aliphatic tail is less well defined (Supplementary Fig. 5) and likely exists in multiple conformations. This structure reveals the molecular basis of TUDCA action as a non-competitive inhibitor, and confirms that tunnel blockade by the bile salt, rather than active site de-stabilization, underlies its inhibitory activity.

These observations reinforce the hypothesis that the tunnel serves as a product exit route^9^; partial inhibition by bile salts further suggests that the tunnel is an alternate but not obligatory exit route for LPA release. It is important to note that, although TUDCA acts as an inhibitor over a wide variety of LPC substrates with different aliphatic tails (Supplementary Fig. 6a–d), TUDCA did not inhibit the hydrolysis of nucleotide substrates (Supplementary Fig. 6e). This further supports the hypothesis that TUDCA acts by blocking LPA exit through the tunnel. Moreover, TUDCA completely inhibited hydrolysis of the fluorescent lysolipid analogue FS3 (ref. 16; *K*_I_=1.4 μM, Supplementary Fig. 6f) in a competitive manner (see the Methods for details). Although it is unknown how ATX binds FS3, it seems likely that one of the two FS3 acyl chains with the attached fluorophore is accommodated in the ATX tunnel, making TUDCA-mediated inhibition for FS3 competitive in nature. We conclude that the tunnel is an allosteric site that binds bile salts to reduce LPA production rates, most likely by slowing down product release.

### Bile salts attenuate ATX function *ex vivo* and *in situ*

**Figure 6:**
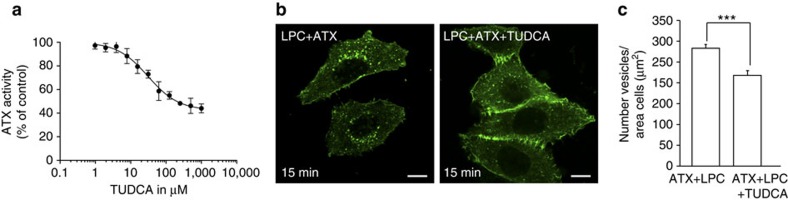
Physiological effects of bile salts on human serum and LPA receptor activation. (**a**) Inhibition of lysoPLD activity is human serum supplemented by TUDCA, measured as released choline by LPC(18:1) hydrolysis. The error bars represent s.e.m. from three different samples. (**b**) Representative confocal images of untreated and TUDCA-treated HeLa cells transfected with GFP-LPA_1_; the number of GFP-LPA_1_-containing endosomes is lower in TUDCA-treated cells. Scale bar, 10 μm. (**c**) Quantification of LPA_1_ internalization shows that TUDCA reduces the number of endosomes compared with untreated cells; error bars correspond to s.e.m. from 27 images in two independent experiments; ***P<0.001 according to Student's *t*-test.

To validate that bile salts act as allosteric inhibitors in a physiological setting, we examined their effect on modulating LPA production in human serum. Consistent with our *in vitro* findings, TUDCA inhibits endogenous ATX activity in human serum *ex vivo* (Fig. 6a) with an apparent IC_50_ of ∼30 μM. Also in serum, TUDCA behaves as a partial inhibitor, leaving ∼40% residual activity. Next, we examined the ability of bile salts to modulate ATX-LPA signalling, by examining their effect on ATX-dependent LPA receptor activation in cultured cells (*in situ*), using agonist-induced LPA1 receptor internalization as readout^17^,^18^. As shown in Fig. 6b,c, at low LPC substrate levels, TUDCA reduces ATX-mediated LPAR1 endocytosis in HeLa cells, consistent with reduced and/or delayed LPA production and delivery.

## Discussion

Our finding that bile salts such as TUDCA and glycochenodeoxycholate act as allosteric inhibitors of ATX lysophospholipase D activity, suggests that ATX may interact with steroids *in vivo*. Under pathological conditions, notably cholestasis, bile salt concentrations can reach >100 μM in the blood stream^19^. Thus, the observed binding constants of bile salts to ATX (∼10 μM) suggest that bile salts are likely to modulate ATX activity under natural conditions. As the lifetime of the ATX:LPA complex is relatively long (tens of seconds^15^) allowing the complex to diffuse over a distance of ∼65 μm before dissociating, bile salt-mediated stabilization of the ATX:LPA complex could enable further spreading of the LPA signal and allow LPA-loaded ATX to interaction with integrin receptors^8^ or heparan proteoglycans^14^ in a more productive manner.

Modulation of ATX activity by bile salts is an intriguing finding given that cholestatic itch is accompanied by a significant increase in serum ATX activity^19^. In addition, intradermal injection of LPA in mice leads to a transient but significant increase in scratch behaviour, strongly suggesting that LPA produced by ATX initiates or potentiates itch^19^. As cholestasis is characterized by accumulation of bile salts in the circulation, this is at first sight contradictory to our findings. However, delaying LPA release by prolonging the lifetime of the ATX:LPA complex, might have stimulatory effects in select cells, such as sensory neurons involved in itch perception.

In the context of cholestatic disease, it is noteworthy that UDCA is often used for the treatment of women with cholestasis of pregnancy (ICP), which develops in ∼1% of pregnant women during the third trimester of pregnancy, to relieve itch^20^. As ATX activity in the serum of these patients is significantly increased and serves as a diagnostic marker^21^, our results suggest that the antipruritogenic effect of UDCA may be attributed, at least in part, to inhibition of plasma ATX activity. This notion is also supported by our finding that TUDCA inhibits serum ATX *ex vivo* (Fig. 6a).

In addition, UDCA is the only FDA-approved drug for the treatment of primary biliary cholangitis, a disorder that leads to progressive cholestasis and is often associated with chronic itch^22^. Moreover, TUDCA has been shown to alleviate symptoms of arthritis in rats^23^; interestingly, ATX has been implicated in the pathogenesis of modelled arthritis^24^. Anti-inflammatory effects of UDCA and its conjugated derivatives have also been recognized in other non-hepatic disorders in which ATX could play a role, such as neurite growth impairment^25^, colitis^26^ and neovascularization^27^. Furthermore, UDCA has been explored as a drug to alleviate symptoms of several neurodegenerative diseases including ALS, Parkinson's disease and Huntington's^28^; although molecular mechanisms that implicate an apoptotic function and ER stress relief have been suggested^29^,^30^, the mechanism of action of UDCA and its derivatives remains an active field of research. The hypothesis that the therapeutic effect of ursodeoxycholates is partially achieved also by unwittingly interfering with ATX function and LPA signalling warrants further investigation.

Various steroidal and non-steroidal agonists of the bile salt receptors TGR5 and FXR are being investigated as candidate drugs for the treatment of chronic liver disease, hepatocellular cancer and extrahepatic inflammatory and metabolic diseases^31^. More specifically developments have targeted obesity, type 2 diabetes, hypertriglyceridaemia and atherosclerosis^32^. In addition, obetichocolic acid is being evaluated in clinical trials as a treatment for non-cirrhotic, non-alcoholic steatohepatitis^33^. The interaction of ATX with bile salts, even regardless of their ability to modulate LPA signalling, might be important for delivering such drugs to their cognate receptors, much as already proposed for ATX and LPA^7^,^9^.

Oxysterols, which clearly bind ATX but did not appear to change ATX activity *in vitro*, using LPC(18:1) as substrate, have emerged as signalling molecules linked to breast cancer pathophysiology^34^. We cannot exclude the possibility that certain oxysterols (or other steroids) might be affecting the processing of other physiologically relevant unsaturated LPC species with longer acyl chains (that are not available for testing).

LPA binds to albumin. How the protein-bound LPA pool, the bio-active LPA pool (which can also be the ATX-bound LPA) and the LPC substrate pool are correlated is poorly understood. Recently, Hla and co-workers have shown that altering the balance of apolipoprotein M and free S1P influences lymphopoiesis and neuroinflammation^35^. By analogy LPA, and steroids might affect the balance between plasma protein and ATX-bound pools of LPA, selectively promoting or inhibiting LPA signalling.

Finally, steroids specific for the ATX tunnel may offer an alternative strategy for developing ATX inhibitors as partial inhibitors might prove of importance. In addition, blocking the ATX tunnel by molecules that slow down LPA release, might even promote LPA signalling, by allowing more efficient delivery of LPA to its receptors.

In conclusion, our study reveals a previously unknown interplay between ATX-mediated LPA production and bile salts acting as allosteric modulators of ATX activity, with potential physiological and clinical implications.

## Methods

### ATX production and crystallization

ATX from rat (rATX) and human (hATX) proteins were produced in HEK 293 Flp-In cells as described previously^36^. The high-resolution crystals that contained 7HCS, rATX (3.5 mg ml^−1^) was first pre-incubated with 23.5 mg ml^−1^ heparin sulphate; 1 μl of this solution was then mixed with 1 μl of reservoir solution containing 20% (w/v) PEG 3350, 0.1 M NH_4_I and 0.3 M NaSCN. After 1 week, the first crystals formed, to finally obtain a crystal of 0.15 × 0.02 × 0.02 mm^3^. This unusually large crystal diffracted to high resolution; although we did not investigate in detail why, these were some of our best-diffracting crystals at the time. We believe that we obtained them through the combination of a good protein preparation (the quality of protein preps often vary), optimizing little practical details of ATX crystallization (as this is done manually there is variation), and reducing the pH close to 6.0 due to the acidic heparin (which we measured at the end in the reservoir solution). For co-crystallization of rATX with TUDCA, TUDCA was first mixed in 10 M excess with rATX (3.5 mg ml^−1^) and incubated for 30 min. For co-crystallization of rATX with TUDCA and LPA(18:1) (Avanti Polar Lipids), rATX (3.5 mg ml^−1^) was first mixed with a 3 M excess of LPA(18:1) and 10 M excess of TUDCA, and incubated for 30 min. In both cases, the best crystals were obtained at room temperature (293 K) in a 24-well optimization screen (18–20% PEG 3350, 0.1–0.4 M NaSCN and 0.1–0.4 M NH_4_I) by mixing 1 μl of protein:inhibitor solution and 1 μl of reservoir solution. All crystals were vitrified in cryoprotectant, which consisted of reservoir solution with the addition of 20% (v/v) glycerol.

### Crystallographic data and methods

Structural studies were performed with rATX. The X-ray diffraction data for the complexes were collected at ESRF on beamlines ID29 (7HCS, TUDCA:LPA) and ID23 (TUDCA), respectively, at 100 K. The crystallographic diffraction data were recorded on a PILATUS detector to resolution of 1.60 Å for the 7HCS complex, 2.20 Å for the TUDCA complex and 1.80 Å for the TUDCA:LPA complex. All data were processed and integrated with XDS^37^ and scaled with AIMLESS^38^. The structures were determined by molecular replacement using PHASER^39^ with the structure of ATX (PDB entry 2XR9 (ref. 8)) as the search model. Model building and subsequent refinement were performed iteratively with COOT^40^, REFMAC5 (ref. 41) and PDB_REDO^42^.

The 7HCS structure was first extensively refined, before we attempted modelling of cholesterol and then 7HCS, as described earlier. For the TUDCA structure, TUDCA was initially modelled in a single conformation in one of the two ATX molecules in the asymmetric unit; after refinement, a second molecule was modelled in the ATX molecule in the second asymmetric unit. After extensive refinement, the TUDCA moiety in the first asymmetric unit was modelled in three different conformations to account for residual difference density. Following that, some neighbouring residues were also modelled in multiple conformations. In the TUDCA:LPA complex, the TUDCA and LPA moieties were modelled directly in the difference density map after initial refinement. Structure validation was carried out by MolProbity, and shows that 97% of all residues where within the Ramachandran plot favoured regions for all three structures, while one residue was an outlier; the clash score and the MolProbity^43^ score for all three structures belong to the 100th percentile. Additional crystallographic parameters and model quality indicators are summarized in Table 1. The structure models and experimental diffraction data were deposited at the PDB under codes 5DLT for 7HCS, 5DLV for the TUDCA complex and 5DLW for the TUDCA–LPA complex.

Structural alignment of the TUDCA structure with the 7HCS structure or the 2XR9 structure performed by RAPIDO^44^, suggested that an optimal alignment should include two rigid bodies: the first consisting of most of the SMB domains (residues 56–100 and 110–120) and a loop of the phosphodiesterase domain (275–284), and the second of the rest of the protein. Both rigid bodies are similar between the two structures (root-mean-square (RMS) deviation 0.5 Å). However, superposing on the second, larger, rigid body shows that the SMB domains have an RMS deviation of 2.3 Å to each other, showing that they moved ‘outwards' to accommodate TUDCA in the tunnel.

### Mass spectrometry analysis

Recombinant hATX preparations or buffer controls were extracted using acidified organic solvents as reported previously for lipid extractions from cells and tissues^45^ with inclusion of deuterium-labelled cholesterol of oxysterol internal standards as detailed below. Samples were evaporated to dryness and reconstituted in methanol for mass spectrometry analysis.

Cholesterol and hydroxysterols were analysed using an AB Sciex 4000-Qtrap hybrid linear ion trap triple quadrupole mass spectrometer in multiple reaction monitoring mode connected to a Shimadzu HPLC. Cholesterol was analysed in positive multiple reaction monitoring mode using a Machery Nagel Nucleodur C8 gravity column, 5 μm, 125 × 2 mm^2^ with a flow rate of 0.5 ml min^−1^. Solvent A consists of 75/25 of methanol/water with 0.5% formic acid and 0.1% 5 mM ammonium formate and solvent B consists of 99/1 of methanol/water with 0.5% formic acid and 0.1% 5 mM ammonium formate. The cholesterol measurements were conducted exactly as reported previously with quantification accomplished by reference to a deuterated internal standard^45^. Oxysterols were measured after dimethylglycine (DMG) derivatization using an Agilent Zorbax Eclipse XDB-C18 column, 5 μm, 4.6 × 150 mm^2^ with a flow rate of 0.5 ml min^−1^. Solvent A was 85% methanol with 5 mM ammonium acetate and solvent B consisted of 80:20:2 of chloroform/water/50 mM ammonium acetate. In brief, we obtained authentic standards for 25-, 24-, 27, 7-α and 4-β OH cholesterol and deuterated 25-OH cholesterol and prepared DMG derivatives exactly as previously described^46^. We obtained product ion spectra from the doubly and singly charged DMG derivatives of these compounds that were essentially identical to those reported previously with some differences in product ion abundances that are likely attributable to differences between our mass spectrometer system and that used by Jiang *et al*.^46^. The DMG derivatives of these authentic standards were used in conjunction with known information reported^46^ to develop mass spectrometry methods that measure product ions that are common to these derivatives and ions that are diagnostic of the particular oxysterols that were then used in selected reaction monitoring mode HPLC-coupled tandem mass spectrometry assays using chromatography methods that could separate these standards. Quantification of oxysterols was accomplished by reference to the deuterated 25-OH cholesterol internal standard as explained below.

Cholesterol was detected in all ATX preparations analysed. Extracted ion chromatograms for two precursor product ion pairs, which were used to detect and quantify the deuterated cholesterol internal standard and ATX-associated cholesterol, are shown in Supplementary Fig. 2, while Table 2 shows representative data from one ATX preparation.

As reported by Jiang *et al*.^46^, bis-DMG derivatives of hydroxylated cholesterol generate doubly charged molecular ions (*m/z*=287.3), which fragment to produce protonated DMG (*m/z*=104.1) and production of singly charged mono-DMG derivative ions (*m/z*=367.2). Further fragmentation/rearrangements of the *m/z*=367.2 ion and its products through processes that are described in detail by Jiang *et al*.^46^ generates ions of *m/z*=232.2 and *m/z*=255.2 that permit discrimination of 7-OH cholesterol from other oxysterols. Accordingly, we established a selected ion-monitoring mode HPLC LC MS/MS assay that monitored these ion pairs. Supplementary Fig. 2 shows extracted ion chromatograms for these four ion pairs identifying 7-OH cholesterol as the major oxysterols associated with the ATX preparation we analysed. Jiang *et al*.^46^ also reported relative differences in signal between the 387.2 and 255.2 ions that discriminate the 7-α and 7-β OH cholesterol epimers. The relative intensities of these ions in the data shown in the figure suggest that the major species is the 7-α epimer, as also suggested by the structural data. Quantification was accomplished by comparing the integrated peak area for the 387.2/104.1 ion pair to that of the corresponding ion pair from the deuterated 25-OH cholesterol internal standard. That ATX preparation analysed contained 1.48 pmol of 7-OH cholesterol. Analysis of extracted ion chromatograms for the precursor and product ions that are common to the bis DMG derivatives of hydroxylated cholesterols with comparison to the retention times of suggested that the preparation contained significantly lower levels of 24-, 25- and 27-OH cholesterols (0.47 pmol).

### Biochemical assays and modelling of kinetic data

All biochemical studies were performed with hATX. ATX lysoPLD activity was measured by choline release from LPC^47^. 20 nM ATX (prepared from HEK 293 Flp-In cells) was incubated with 150 μM LPC(18:1) in a final volume of 100 μl buffer containing 50 mM Tris, pH 7.4, 0.01% Triton X-100, 50 mM CaCl_2_, 1 U ml^−1^ choline oxidase, 2U ml^−1^ horseradish peroxidase, 2 mM homovanilic acid (HVA). The relative amount of released choline was measured by HVA fluorescence in a 96-well plate (Corning). Fluorescent intensity was determined at *λ*_ex_/*λ*_em_=320/450 nm every 30 s for 90 min with a Fluorostar plate reader (BMG Labtech). Data analysis was performed using GraphPad Prism.

Nucleotide phosphodiesterase activity was measured using pNP-TMP (Sigma-Aldrich) as substrate in 50 mM Tris (pH 7.8), 140 mM NaCl, 5 mM KCl, 1 mM CaCl_2_, 1 mM MgCl_2_, 1 mg ml^−1^ fatty acid-free BSA. After addition of recombinant ATX, the amount of liberated *para*-nitrophenolate (pNP) was determined by the absorbance at 405 nm. The relative amount of released pNP was determined by the absorbance at 405 nM every 30 s for 90 min with a Fluorostar plate reader (BMG Labtech). Data analysis was performed using GraphPad Prism.

ATX activity against the FS3 substrate^16^ was measured by incubating 20 nM ATX with the indicated range of FS3 in a final volume of 50 μl in 50 mM Tris buffer (pH 7.4) containing 0.2 mg ml^−1^ fatty acid-free BSA. Fluorescent intensity was determined at *λ*_ex_/*λ*_em_=485/520 nm every 30 s for 90 min with a Fluorostar plate reader (BMG Labtech).

The experiments for determining IC_50_ for different bile salt inhibitors were performed by adding the named bile salt and performing serial threefold dilutions; for each bile salt concentration, the velocity of the reaction was monitored as a function of time in the lysoPLD assay as described above. The linear part of the reaction velocity was visually estimated (typically between 10 min and 1 h) and the observed fluorescence signal (described above) as a function of time was modelled for all inhibitor concentrations simultaneously using the formula:


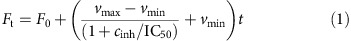


where *F*_t_ is the observed fluorescence signal relative to the choline product at each time point (*t)*, *F*_0_ is the background fluorescence signal at the start of each measurement, *v*_max_ and *v*_min_ are the fitted values for the minimum and maximum relative velocity (common for all inhibitor concentrations) and *c*_inh_ is the corresponding inhibitor concentration for each of the experiments.

At the same time, we have also derived an observed velocity *(V)* by linear regression of the fluorescence signal for each inhibitor concentration and modelled that as a function of inhibitor concentration using the formula:


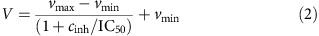


for cross-checking the result of equation (1) and for creating the inhibition graphs presented in various figures.

For initial comparison between competitive, uncompetitive and non-competitive inhibition by TUDCA, using the LPC and the FS3 assays, we measured the relative velocity for increasing LPC or FS3 substrate concentrations, for no-inhibitor and for three different inhibitor concentrations. The relative velocity for each data point was measured by linear regression as described above, and a Michaelis–Menten curve was created for each inhibitor concentration. We then used the following equations to describe each mode of inhibition:


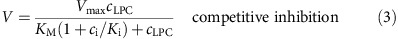



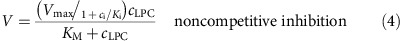



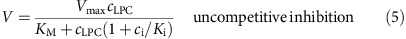


where *V* is the observed velocity and *c*_LPC_ is the corresponding LPC concentration for each data point, and *c*_i_ is the inhibitor concentration for each curve; *K*_M_ and *V*_max_ are the Michaelis–Menten constants and the estimated maximum velocity in a relative scale, and *K*_i_ is the inhibition constant. Note that in equation (5) *K*_i_ denotes the product of *K*_i_ (which is very high as the inhibitor actually does not bind alone) and a constant that defines the apparent *K*_i_ of the ternary enzyme–substrate–inhibitor complex.

The preferred model was estimated using pairwise comparisons and we chose the best-fitting model utilizing Akaike's Informative Criteria to select the model most likely to have generated the data. For the LPC hydrolysis data, the competitive model had <0.01% probability to be correct when compared with either the noncompetitive or the uncompetitive models, whereas the noncompetitive model showed a moderate preference (75%) over the uncompetitive model (25%). For the FS3 data, the competitive model had a probability of more than 99.99% to be correct compared with other models.

For understanding the inhibition of LPC hydrolysis by TUDCA in further detail, we used a partial mixed inhibition model defined by the equation


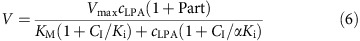


where in addition to previous definitions that can be derived from equations (4–6), we define Part as the partiality of the inhibition and *α* as an estimated constant that determines mechanism: if *α=1*, the formula becomes identical to equation (4) and the inhibition mode is noncompetitive; if *a* is very large, then the model approaches a competitive model equation (3); if *a* is very small but greater than zero, the model approaches an uncompetitive model.

For more robust fitting, we also did an experiment where we varied the inhibitor concentration for a fixed amount of substrate, which we modelled using an equation similar to equation (2):


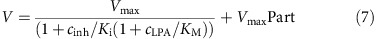


equations (6) and (7) were fit simultaneously, each in the data set(s) they describe (with a relative scale factor applied to (7) to account for experiment differences), with the parameters that are determined in both experiment (*K*_M_, V_max_*, K*_i_, Part) constrained to have identical values between experiments. This procedure allowed a more robust fit to determine all parameters from all available experiments. The value of *α* refined to 2.3, suggesting a noncompetitive inhibition model, where TUDCA binds to ATX both in the absence and in the presence of LPC (or LPA), albeit with a very modest twofold preference for the substrate-free protein.

All modelling was performed using the regression analysis tools within the Graphpad/Prism software. The values of all derived parameters indicate mean values as obtained by nonlinear regression analysis using the formulas listed above; triplicate data were used as separate observations during the fit; ±values indicate the standard error of the mean.

### Effects of bile salts in the *ex vivo* activity of ATX

An ATX choline release activity assay (see above for details) was done with three different sera from normal pregnant women (informed consent was obtained from all human participants; study protocol approved by the Medical Ethical Committee of the Amsterdam Medical Center). We chose those because ATX activity is relatively high without increased serum levels of bile salts^21^. In place of purified ATX, 20 μl of serum were added in a total assay volume of 150 μl), aiming to dilute the serum as little as possible. Before starting the assay, the serum was pre-incubated with each indicated TUDCA concentration (1:2 dilution) for 5 min. The indicated TUDCA concentration is the final concentration in the assay.

### LPAR internalization assay

HeLa cells (obtained from American Type Culture Collection) were grown in DMEM supplemented with 10% fetal bovine serum at 37 °C under 5% CO_2_. For LPA_1_ internalization studies, HeLa cells were transient transfected with GFP-LPA_1_ plasmid using X-tremeGene 9 (Roche) reagent according to the manufacturer's instructions. At 10 h after transfection, the cells were rinsed with SF-DMEM and incubated in the same medium for 16–24 h before further treatments. Cells were treated with 0.1 μM LPC and 5 nM ATX or with 0.1 μM LPC and 5 nM ATX and 500 μM of TUDCA for 15 min. Coverslips were fixed with 4% paraformaldehyde for 10 min, washed twice with PBS and mounted using Immu-Mount. Slides were examined on a Leica TCS-SP5 confocal microscope (× 62 objective) and analysed using ImageJ software.

### Cell culture and reagents

HEK 293 Flp-In (purchased from Invitrogen) and HeLa cells were maintained in DMEM (Gibco, Life Technologies) supplemented with 10% FCS (HyClone, Thermo Scientific) 2 mM glutamine, 100 U ml^−1^ penicillin and 100 μg ml^−1^ streptomycin (Gibco, Life Technologies). Choline oxidase, HVA, horseradish peroxidase fatty acid-free BSA, Dexamethason, PEG3350, NaSCN, NH_4_I, pNP-TMP (*p*-nitrophenyl thymidine 5′-monophosphate), taurochenodeoxycholic acid, taurodeoxycholic acid, glycochenodeoxycholic acid, ursodeoxycholic acid and LPA were purchased from Sigma-Aldrich. LPC and 7α,25-dihydroxycholesterol from Avanti polar lipids. FS3 (LysoPLD/ATX substrate) from Echelon and TUDCA from Calbiochem.

## Additional information

**Accession code**: The crystal structures have been deposited in the Protein Data Bank with accession codes: 5DLT, 5DLV, 5DLW.

**How to cite this article:** Keune, W.-J. *et al*. Steroid binding to Autotaxin links bile salts and lysophosphatidic acid signalling. *Nat. Commun.* 7:11248 doi: 10.1038/ncomms11248 (2016).

## Supplementary Material

Supplementary Information Supplementary Figures 1-6

## Figures and Tables

**Figure 1:**
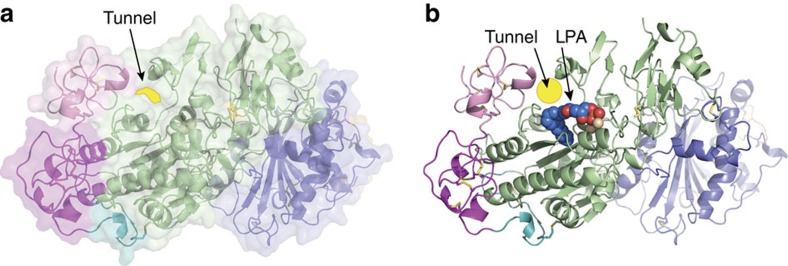
The tunnel of ATX. (**a**) A cartoon representation of the domain structure of ATX combined with a transparent surface highlighting the position of tunnel; the SMB domains are coloured in pink and magenta, the PDE domain in green, the NUC domain in blue, the lasso loop wrapping around the NUC domain in orange and the short loop connecting the SMB domains to the PDE domain in cyan; the zinc ions are shown as spheres; a yellow background highlights the tunnel site, (**b**) the same cartoon model, with bound LPA18:1 (PDB:3NKP) and a yellow circle designating the tunnel site. All structural images were generated using PyMOL (http:// www.pymol.org) or CCP4mg^48^.

**Table 1:** Crystallographic data.

	**ATX+7HCS**	**ATX+TUDCA**	**ATX+LPA+TUDCA**
PDB identifier	5DLT	5DLV	5DLW
*Data collection*			
Space group	P1	P1	P 2_1_ 2_1_ 2_1_
*Cell dimensions*			
*a*, *b*, *c* (Å)	53.67, 63.48, 70.72	62.88, 77.83, 92.44	63.20, 93.05, 149.68
*α*, *β*, *γ* (°)	98.72, 105.80, 99.97	83.84, 79.29, 77.08	90,90,90
Resolution (Å)*	44–1.60 (1.63–1.60)	47–2.00 (2.03–2.00)	46–1.80 (1.83–1.80)
*R*_merge_ (%)	7.5 (59.8)	11.5 (122)	15.2 (240)
*CC*_*1/2*_	1.00 (0.44)	0.99 (0.34)	1.00 (0.26)
Average *I*/σ*I*	6.7 (0.8)	5.6 (0.8)	8.6 (1.0)
Completeness (%)	92.1 (88.9)	97.9 (92.0)	99.8 (99.3)
Redundancy	2.0 (1.9)	3.6 (3.5)	5.9 (5.8)
			
*Refinement*			
Resolution (Å)	44–1.60	47–2.00	44–1.80
No. of reflections	99938	108586	78197
*R*_work_/*R*_free_	17.1/19.6	19.3/22.7	18.8/21.6
No. of atoms†			
Protein+carbohydrates	6542	12896	6399
Ligand+metal ions	34	224	68
Waters and others	613	694	341
B-factors (Å^2^)			
All	29	36	33
Protein+carbohydrates	29	36	33
Ligand+metal ions	26	39	56
Water and others	34	35	35
R.m.s. deviations‡			
Bond lengths (RMSZ/RMSd)	0.461/0.013	0.483/0.010	0.487/0.010
Bond angles (RMSZ/RMSd)	0.658/1.450	0.644/1.435	0.664/1.466

ATX, Autotaxin; LPA, lysophosphatidic acid; PBD, Protein Data Bank; RMS, root-mean-square; TUDCA, tauroursodeoxycholate; 7HCS, 7α-hydroxycholesterol.

^*^Highest resolution shell is shown in parenthesis.

^†^Alternate conformations are counted as multiple atoms.

^‡^As given by REFMAC for all bonds and angles.

**Table 2:** Mass spectrometry analysis of ATX.

	**pmol**	**Ratio**
**ATX**	**2.4**	**1.0**
Cholesterol	0.35	0.15
Total DMG hydroxycholesterol	1.95	0.81
7-α Hydroxycholesterol	1.48	0.62

ATX, Autotaxin.
