# Supplementary material for: Steroid binding to Autotaxin links bile salts and lysophosphatidic acid signalling
Source: Nat Commun. 2016 Apr 14;7:11248. doi: 10.1038/ncomms11248 (PMC4834639; doi:10.1038/ncomms11248)
Supplement: Supplementary Information — Supplementary Figures 1-6 [file ncomms11248-s1.pdf]

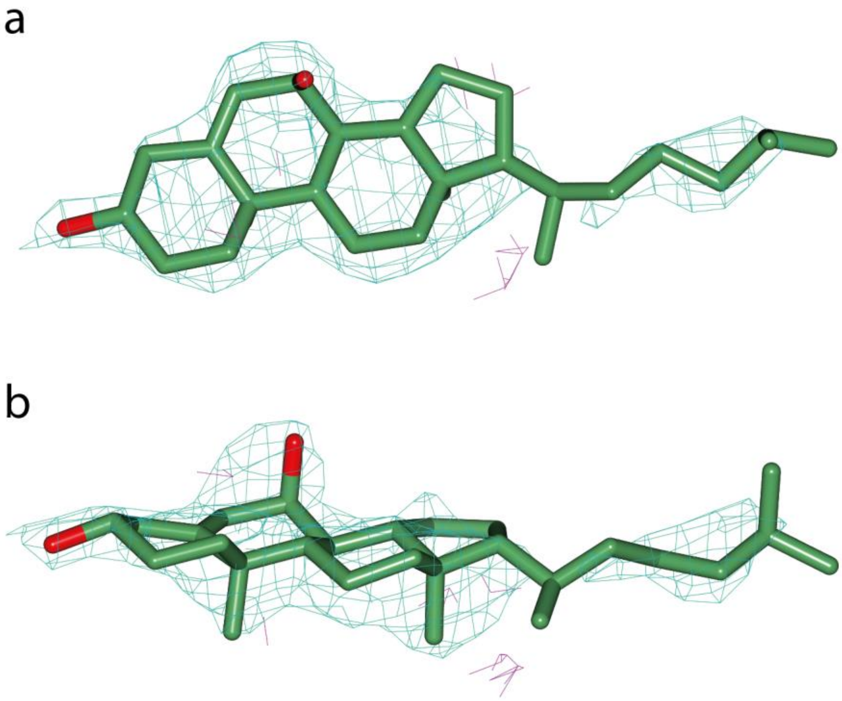

Supplementary Figure 1 | Oxysterol is bound in the native rATX structure (2XR9)

(a,b) The difference density (mF_o-DF_c) that was previous un-interpreted for the 2XR9 entry, is shown at 2.5 *rms* as a green wireframe model; a model for 7HCS shows an excellent fit to the density.

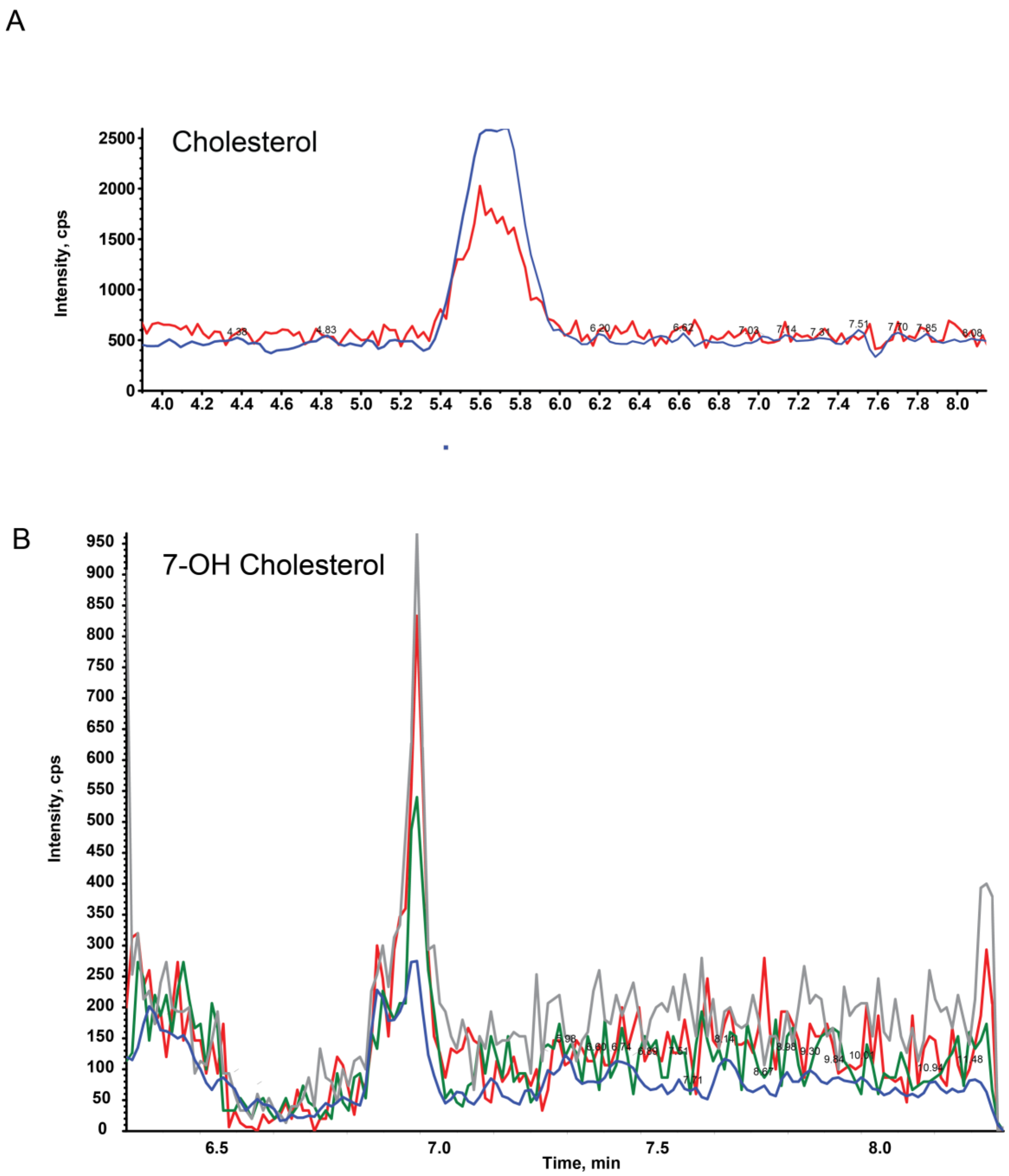

Supplementary Figure 2 | Mass spectrometry analysis of bound sterols

Lipids extracted from recombinant ATX were analyzed by HPLC electrospray ionization tandem mass spectrometry in selected ion monitoring mode. **(a)** shows an extracted ion chromatogram for two precursor product ion pairs m/z 369.4/147.1 (red) and 369.4/109.1 (blue) that are specific for cholesterol. **(b)** an extracted ion chromatogram for four precursor product ion pairs at a retention time corresponding to that of authentic 7-OH cholesterol. The ions monitored are m/z 287.3/367.2 (grey), m/z 367.2/104.1 (red) m/z 367.3/232.2 (green) and m/z 367.2/255.2 (blue).

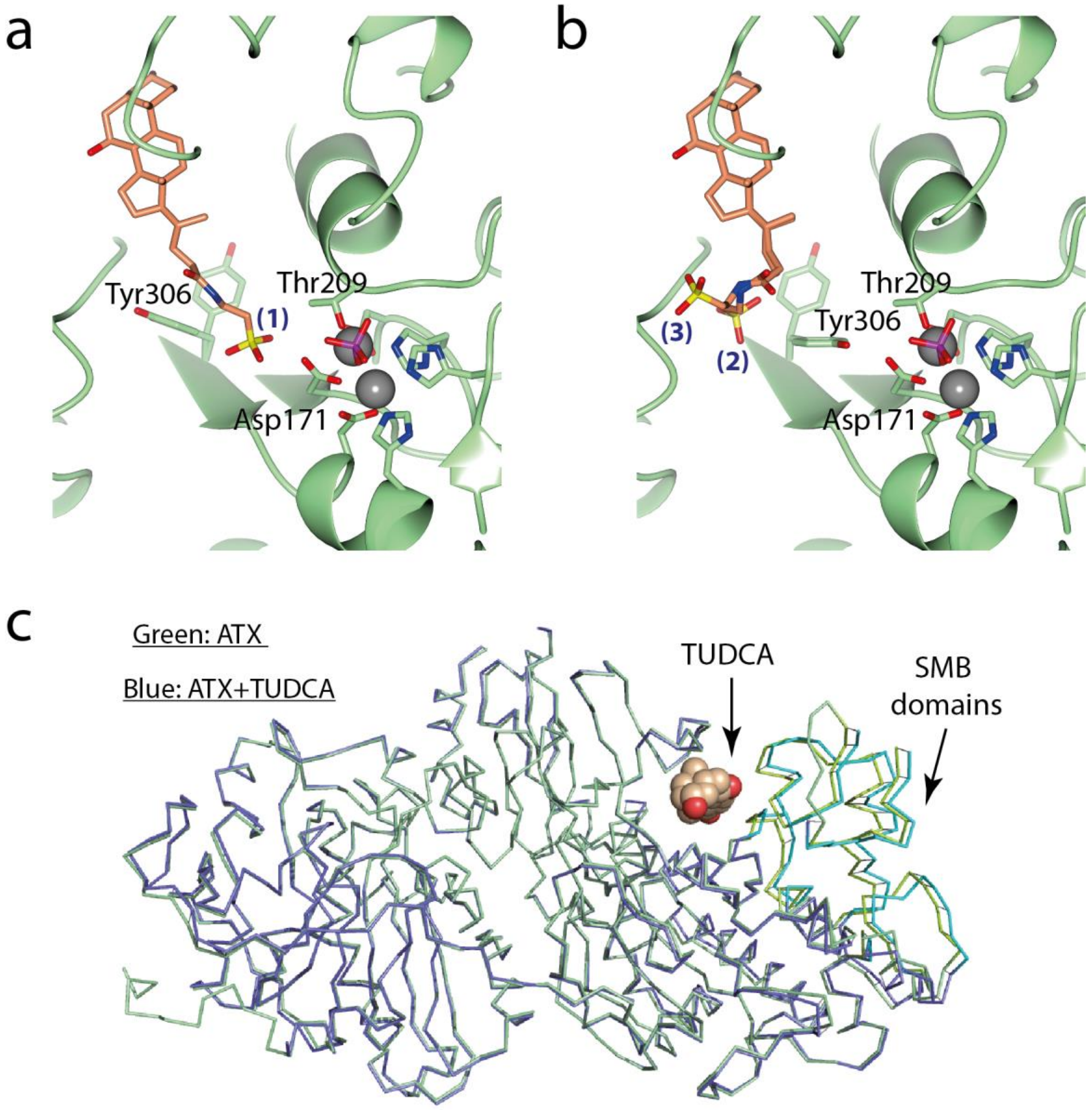

Supplementary Figure 3 | Triple conformation for TUDCA and movement of SMB domains

(a, b) Models of the three finally modelled conformations of the acidic tail of TUDCA in the tunnel (one conformation shown is panel a and the remaining two conformations are shown in panel b), showing the active site with the Thr209 nucleophile, the zincs (shown as grey spheres) and the residues coordinating the zinc atoms; the three different conformations of the tail group are correlated with a displacement of Tyr306, which in some conformations hydrogen bonds to the zinc coordinating Asp171, possibly destabilizing the catalytic site conformation. **(c)** A superposition of the C_a trace of "native" ATX (PDB:2XR9) and ATX bound to TUDCA (green); the movement of the SMB domains to the right (shown in lighter green and blue), widening the tunnel, is apparent.

Supplementary Figure 4 | Various sterols have no effect in LPA hydrolysis by ATX

(a-c) Inhibition of lysoPLD ATX activity measured as released choline by LPC (18:1) hydrolysis in the presence of different sterols. The activity is normalized and error bars represent *sem* from triplicate experiments.

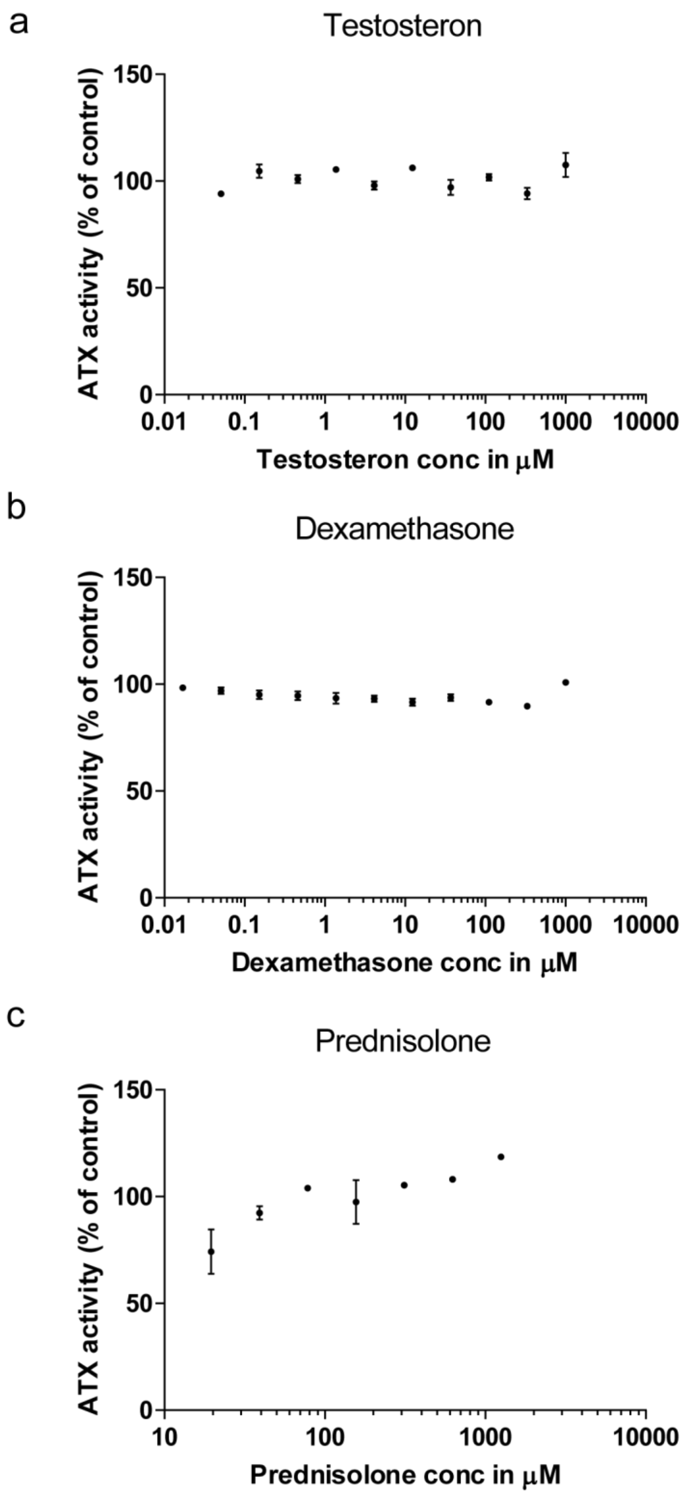

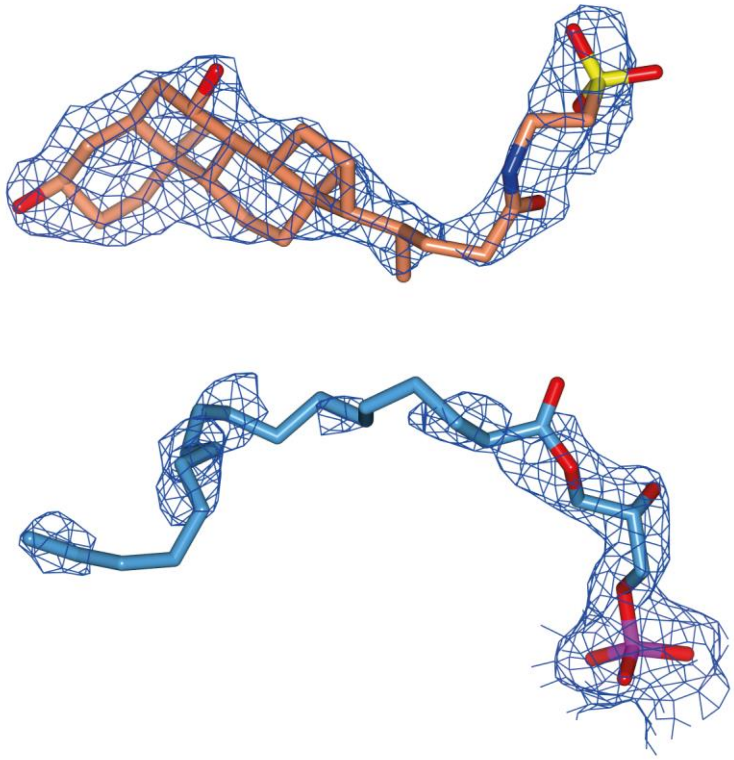

Supplementary Figure 5 | Experimental density for TUDCA and LPA(18:1) bound to ATX

The 2mF_o-DF_c map is shown at 1.0 *rms* as a blue wireframe model; while the TUDCA moiety and the glycerol of the LPA(18:1) are clear, the aliphatic tail of the LPA has less clear density, most likely indicating that it adopts multiple conformations in the structure.

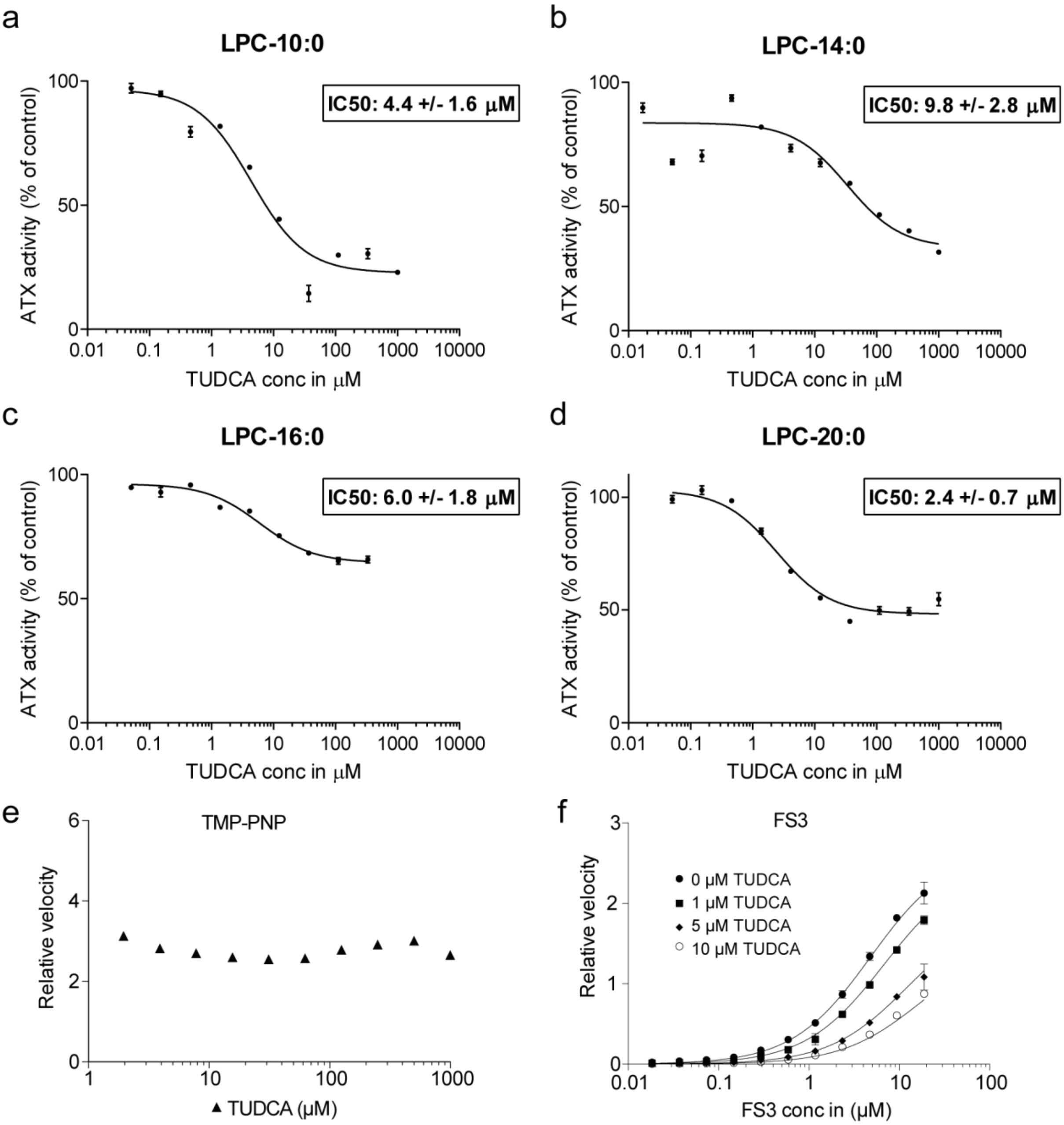

Supplementary Figure 6 | Inhibitory effect of TUDCA in various ATX substrates pNP-TMP and FS3 substrates

(a-d) Inhibition of lysoPLD ATX activity by TUDCA, measured for different LPC species. The activity is normalized. **(e)** ATX activity measured by pNP-TMP hydrolysis against increasing TUDCA concentrations, shows no signs of inhibition. **(f)** ATX activity measured by FS3 hydrolysis (artificial ATX substrate) without and with three concentrations of TUDCA against increasing concentrations of the FS3 substrate. The mode of inhibition of FS3 fits well to a competitive model (see Methods for details). For all panels, the plots were generated using GraphPad/Prism software, and error bars represent *sem* from triplicate experiments.
